# Ameliorative effect of *Gastrodia elata* Blume extract on inflammation in a rat model of osteoarthritis

**DOI:** 10.1186/s42826-026-00297-3

**Published:** 2026-10-21

**Authors:** Yeon Su Lee, Da Eun Jung, Ji Won Seo, Hyeon Jeong Na, Jeong Su Park, Jin Woo Hong, Hyeon Ju Lee, Jae-Ho Shin

**Affiliations:** Department of Biomedical Laboratory Science, Graduate School, Eulji University, Seongnam, Gyeonggi-do 13156 Republic of Korea

**Keywords:** *Gastrodia elata* Blume, Osteoarthritis, Anti-osteoarthritic effects, Anti-inflammatory effects

## Abstract

**Background:**

Osteoarthritis (OA) is characterized by progressive articular cartilage degeneration, in which inflammatory mediators drive MMP-mediated matrix breakdown and chondrocyte apoptosis, ultimately leading to pain and functional impairment. Although often described as a “wear-and-tear” disease, increasing evidence highlights immune/inflammatory signaling as a key contributor to OA progression. Gastrodia elata Blume (GEB) has reported anti-inflammatory and immunomodulatory activities, but its preventive potential in OA remains unclear. This study evaluated GEB extract in a widely used monosodium iodoacetate (MIA)-induced OA rat model. Male Sprague–Dawley rats (5 weeks old) were assigned to CON, OA, OA + L-GEB, and OA + H-GEB groups; GEB was administered orally once daily for 5 weeks, and OA was induced by intra-articular MIA (3 mg/50 µL) at day 21.

**Results:**

GEB improved gross cartilage surface appearance and histopathological outcomes (HE, Safranin O, Alcian blue; Mankin and OARSI scores), showing reduced cartilage damage and better preservation of proteoglycan staining compared with the OA group. Consistently, GEB decreased IL-6, MMP-13, and cleaved caspase-3 in cartilage (IHC) and reduced TUNEL-positive cells, indicating suppression of inflammatory signaling and chondrocyte apoptosis. RT-qPCR further showed downregulation of NF-κB, IL-6, MMP-9, MMP-13, and TIMP-1 mRNA levels in GEB-treated groups versus OA.

**Conclusions:**

GEB extract attenuated inflammatory and catabolic responses and inhibited chondrocyte apoptosis in MIA-induced OA, supporting its potential as a prophylactic strategy for localized inflammatory pathology such as osteoarthritis.

**Supplementary Information:**

The online version contains supplementary material available at https://doi.org/10.1186/s42826-026-00297-3.

## Background

*Gastrodia elata* Blume (GEB) is a renowned herb in traditional Chinese medicine and has garnered significant attention for its diverse biological activities, including anti-inflammatory, immunomodulatory, antioxidant, and neuroprotective properties [1–7]. Notably, the immunomodulatory potential of GEB has been extensively investigated, demonstrating its capacity to attenuate inflammation and oxidative stress in rodent models [8–13]. GEB is composed of multiple bioactive constituents, with gastrodin (GAS) and 4-hydroxybenzyl alcohol (HBA) being the principal components [7, 10, 14]. GAS is known for its anticonvulsant and anti-inflammatory properties. Similarly, HBA has demonstrated efficacy in treating epilepsy, tetanus, and other neurological diseases [7, 8, 14]. Collectively, these findings suggest that GEB may modulate inflammatory responses through its anti-inflammatory and immunomodulatory properties [15, 16]. Notably, these effects are achieved by downregulating the expression of pro-inflammatory cytokines and regulating the activity of key enzymes and immune cells [4, 17]. Despite its well-documented systemic effects, the preventive potential of GEB in localized inflammatory diseases such as osteoarthritis (OA) remain largely unexplored.

OA is the most prevalent form of arthritis, with its prevalence in the elderly population nearly doubling over the past 25 years [18, 19]. OA is characterized by the progressive degeneration of articular cartilage, which manifests clinically as persistent knee pain and gait disturbances [20–24]. Although wear and tear have long been considered the primary cause of OA [25], recent findings emphasize the role of inflammatory signaling pathways [26–29]. Elevated pro-inflammatory cytokines contribute to cartilage degradation, exacerbating disease progression [30, 31].

To replicate these pathological features, the monosodium iodoacetate (MIA)-induced OA model has been widely adopted in preclinical research. MIA inhibits glyceraldehyde-3-phosphate dehydrogenase (GAPDH), a key enzyme in the relevant pathway, disrupting cellular respiration and impairing its function [32–34]. MIA-induced metabolic disruption triggers the release of damage-associated molecular patterns (DAMPs), which interact with pattern recognition receptors (PRRs) on synoviocytes and chondrocytes, initiating inflammatory cascades [35]. These cascades promote the expression and secretion of pro-inflammatory mediators, including cytokines and chemokines, disrupting the equilibrium between matrix metalloproteinases (MMPs) and tissue inhibitors of metalloproteinases (TIMPs) [36–42]. Additionally, this proteolytic imbalance promotes chondrocyte apoptosis, compromising cartilage integrity and accelerating cartilage degradation. These pathological features closely resemble those of human OA, supporting the relevance of the MIA-induced model in preclinical research [40, 43].

Currently available therapeutic strategies for OA include non-steroidal anti-inflammatory drugs (NSAIDs) for pain management and immunosuppressants to regulate the inflammatory response, providing symptomatic relief without fully restoring damaged cartilage [23, 32]. However, these treatments have limited effectiveness and are associated with adverse effects such as gastrointestinal complications and immunosuppression [23, 32]. Therefore, developing novel therapeutic agents that modulate inflammatory pathways while targeting cartilage preservation is imperative. Additionally, the limitations of current therapies for localized inflammatory conditions emphasize the need for novel therapeutic strategies.

Therefore, we investigated the prophylactic effects and potential mechanisms of GEB extract in inflammatory and immune responses in a rodent model of OA using various biochemical and molecular assays, including immunohistochemistry, histopathological assay, and western blotting. Overall, it is anticipated that this study will provide relevant information on the protective mechanisms of GEB extracts in inflammatory diseases such as OA.

## Methods

### Preparation of GEB extract

The GEB was provided by the Rural Development Administration (Jeonju-si, Jeollabuk-do, Korea) and prepared for experimental use through a series of processing steps. First, the GEB was sliced, dried, and coarsely ground. For pre-treatment, it was treated with α-amylase-termamyl 2x at 95–98 °C for 2 h to facilitate extraction. The extraction process was then performed by boiling the pre-treated GEB in Distilled water (DW) at 96 °C for 4 h using 10 times its weight in DW. This process was repeated four times, and the resulting extracts were combined. To ensure microbial safety, the combined extract was sterilized at 95 °C for 90 min, lyophilized, and powdered. The lyophilized powder was further sterilized by irradiation for long-term stability. Subsequently, the extract was subjected to High-performance liquid chromatography-diode array detector (HPLC-DAD) analysis for basic phytochemical characterization, which identified GAS and HBA as the primary active compounds (Tables 1 and 2). The obtained GEB powder was then mixed with DW to prepare fresh doses, at a concentration of 400 mg/mL (low dose) or 800 mg/mL (high dose), for weekly administration. These doses were stored at -4 ℃ until use.

**Table 1:** HPLC-DAD instrumental analysis conditions for the analysis of GEB extract

LC (Liquid chromatography)		Agilent 1100 series						
Column		Capcellpak C18 UG1200 (4.6 × 250 nm, 5 μm)						
Mobile phase		A: Water: ACN = 99:1 B: ACN						
Gradient mode		Time (min)	0	15	20	25	25.1	30
		B (%)	0	0	90	90	90	0
Flow		1.2 mL/min						
Injection volume		10 µL						
Detector	DAD detector	270 nm						

**Table 2:** GAS and HBA contents of low- and high-concentration GEB extract confirmed using HPLC-DAD

Contents (mg/L)	GAS	HBA
L-GEB	456.64	69.4
H-GEB	913.28	138.8

### Chemicals and reagents

MIA was purchased from Sigma Aldrich (St. Louis, MO, USA) and dissolved in sterilized saline before use. Immunohistochemical (IHC) assay was performed using primary antibodies, including interleukin-6 (IL-6; ab6672, Abcam, Cambridge, UK), matrix metallopeptidase-13 (MMP-13; ab39012, Abcam), and cleaved caspase-3 (9661S, Cell Signaling Technology, Massachusetts, USA) and an ABC-horseradish peroxidase (HRP) (peroxidase, rabbit IgG) kit (PK6101, Vector Laboratories, Burlingham, CA, USA). Terminal deoxynucleotidyl transferase dUTP nick end labeling (TUNEL) was performed to examine cell death in cartilage sections using the In Situ Cell Death Detection Kit, POD (cat #11 684 817 910, Roche, Basel, Switzerland).

### Animals

Five-week-old male Sprague-Dawley (SD) rats (Samtako Bio, Osan, Korea) were housed under standard conditions (12-hour light/day cycle with 22 ± 2 °C temperature and 50 ± 10% humidity) and had free access to commercial food (Purina CO.) and water. The water and food intake were measured twice a week for 35 days. The experimental protocol was approved by the Institutional Animal Care and Use Committee of Eulji University (EUIACUC 24 − 01).

### Experimental design

After 7 days of adaptation, rats were randomly divided into four groups: control (CON), OA, OA + low-GEB (OA + L-GEB), and OA + high-GEB (OA + H-GEB) groups. Rats in the CON and OA groups were orally administered DW, whereas those in the OA + L-GEB and OA + H-GEB groups received 10 mL/kg of GEB extract (L-GEB contained 456.64 mg/L of GAS and 69.4 mg/L of HBA; H-GEB contained 913.28 mg/L of GAS and 138.8 mg/L of HBA). The selected doses of GEB extract were based on previous studies demonstrating no observable toxicity at these levels [44, 45]. In this study, GEB was administered before MIA injection; therefore, the experimental design was considered preventive rather than therapeutic.

To induce OA, MIA (3 mg/50 µL in saline) was injected intra-articularly into the left knee joint of the rats in the OA, OA + L-GEB, and OA + H-GEB groups at 21 days GEB extract administration. Rats in the CON group did not receive MIA injection, as these rats served as the healthy control group. The extract was carefully diluted to achieve the specified concentrations for the L-GEB and H-GEB groups.

All treatments were administered for five weeks. Oral administration of the treatments was performed once daily, every morning, during the experimental period (Supplementary Fig. 1). At 24 h after the last administration, the rats were fasted overnight and anesthetized with isoflurane (Halocarbon, Georgia, USA). Additionally, tibial cartilage from the left knee joint was fixed and decalcified for histological analysis, immunohistochemistry, and TUNEL assay. Moreover, femoral cartilage from the left knee was frozen for quantitative reverse transcription polymerase chain reaction (RT-qPCR).

### Cartilage sample collection for histological analysis

Briefly, tibial cartilage samples were collected in 50 mL tubes and fixed overnight in 10% neutral buffered formalin (NBF) under gentle agitation for histological analysis. Thereafter, the samples were decalcified for 3 days using the RDO Rapid Decalcifier (Apex Engineering Products Corporation, IL, USA), washed in running water, and transferred back to 10% NBF for 1 day. Finally, the samples were dehydrated using ascending ethanol series (70, 80, 90, and 100%), cleared in xylene, and embedded in paraffin.

### Histological assays

Briefly, the cartilage samples were prepared as previously described, cut into 4 μm thick sections using a microtome (HM3405E, Thermo Fisher Scientific, Waltham, MA, USA), deparaffinized in xylene, and hydrated using ethanol series (anhydrous to 70% ethanol). Thereafter, the sections were stained with Harris hematoxylin for 30 s and eosin Y for 30 min to examine changes in cartilage structure. Additionally, the cartilage was stained with Safranin O and alcian blue to assess the extent of proteoglycan loss in the cartilage matrix. Safranin O staining was performed with Weigert’s iron hematoxylin for 5 min, followed by differentiation in 1% acid-alcohol for 2 s. Background staining was performed with 0.02% fast green (F7242-5G, Sigma) for 1 min to enhance contrast, followed by proteoglycan staining with 1% safranin O (S2255-25G, Sigma) for 30 min. For alcian blue staining, the sections were deparaffinized, hydrated, treated with sulfated glycosaminoglycans (GAGs) for 30 min, and stained with alcian blue solution prepared from alcian blue 8GX powder (A3157, Sigma) and adjusted to pH 1.0 using hydrogen chloride (HCl). Thereafter, the sections were counterstained with Harris hematoxylin for 5 min to visualize the nuclei. Cartilage damage and proteoglycan loss were assessed according to the criteria described in Tables 3 and 4. Notably, the observed discrepancy in staining regions is attributable to minor positional variations introduced during the sectioning and staining processes.

**Table 3:** Modified Mankin score for the microscopic evaluation of the articular cartilage of rats with osteoarthritis

Criteria	Score	Histological finding
Structure	0	Smooth intact surface
	1	Slight surface irregularities
	2	Pannus/Surface fibrillation
	3	Clefts into the transitional zone
	4	Clefts into the radial zone
	5	Clefts into the calcified zone
	6	Total disorganization
Cells	0	Uniform cell distribution
	1	Diffuse cell proliferation
	2	Cell clustering
	3	Cell loss
Tidemark integrity	0	Intact
	1	Crossed by blood vessels

**Table 4:** OARSI score of the articular cartilage of rats with osteoarthritis

Grade	Key feature	Stage % involvement (surface, area, volume)			
		Stage 1 (< 10%)	Stage 2 (10–25%)	Stage 3 (25–50%)	Stage 4 (> 50%)
Grade 0	Normal cartilage	0	0	0	0
Grade 1	Surface intact	1	2	3	4
Grade 2	Surface focal discontinuity	2	4	6	8
Grade 3	Vertical fissures (clefts)	3	6	9	12
Grade 4	Cartilage erosion	4	8	12	16
Grade 5	Denudation	5	10	15	20
Grade 6	Deformation	6	12	18	24

* Score = grade x stage

### Histological analysis of immunohistochemistry

For IHC, paraffin sections  (4 μm) were mounted on silane-coated slide glasses (Matsunami, Japan). After deparaffinization and rehydration, antigen retrieval was performed using one of three methods: boiling in citrate buffer with a microwave for 10 min, incubation with 1X PBS containing proteinase K (25530015, Invitrogen) at 37 °C for 30 min, or treatment with trypsin at 37 °C for 30 min. The primary antibodies, including IL-6 (diluted 1:800, ab6672, Abcam), MMP-13 (diluted 1:1,000, Abcam), and cleaved-caspase 3 (diluted 1:400, Cell Signaling Technology) were incubated overnight at 4 °C. The slides were visualized with DAB (34002, Thermo). Finally, the slides were counterstained with Mayer’s hematoxylin, and examined under a light microscope (Olympus optical co.). For quantitative analysis, IL-6- and cleaved caspase-3-positive cells were counted in the articular cartilage. MMP-13 staining was evaluated qualitatively because its immunoreactivity was broadly distributed in the cartilage matrix and surface region, making reliable positive-cell counting difficult.

### TUNEL assay

TUNEL assay was performed to assess apoptosis-associated DNA fragmentation in cartilage sections using an in situ cell death detection kit, POD (11684817910, Roche). Briefly, deparaffinized sections were treated with proteinase K (20 µg/mL, 37 °C, 30 min), quenched with 3% H₂O₂ in methanol (30 min), and blocked with PBS containing normal goat serum and BSA (1 h). Sections were incubated with TUNEL reaction mixture (37 °C, 60 min, dark), followed by converter-POD (37 °C, 30 min). Signals were developed with DAB and counterstained with Mayer’s hematoxylin. TUNEL-positive cells were blindly counted under a light microscope.

### RNA isolation from cartilage and RT-qPCR

Cartilage was scraped from the femora using sterilized scissors, collected in cryotubes, snap-frozen in liquid nitrogen, and stored at − 80 °C until analysis. Total RNA was extracted using TRIzol reagent (Invitrogen), followed by 2-propanol precipitation and a 75% ethanol wash, and then resuspended in DEPC-treated water; RNA purity was confirmed by A260/A280 (average ~ 2.0). cDNA was synthesized from 1 µg RNA using the PrimeScript 1st Strand cDNA Synthesis Kit (Takara). qPCR was performed on a QuantStudio 1 Real-Time PCR System (Thermo) using PowerUp SYBR Green Master Mix (Thermo) to quantify mRNA levels of NF-κB, IL-6, IL-1β, MMP-9, MMP-13, and TIMP-1, with GAPDH as the internal control. Amplification was run for 40 cycles and specificity was verified by melting-curve analysis. All reactions were performed in triplicate, and relative expression was calculated using the ΔΔCt method. The sequences of the primers are presented in Table 5.

**Table 5:** Primer sequences used for RT-qPCR assay

Gene	Primer sequence	Product amplified (bp)
NF-κB	F: ACAGATACCACTAAGACGCACCC	237
	R: AATGGCTTGCTCCAGGTCTCG	
IL-6	F: AAAGCCAGAGTCATTCAGAGC	106
	R: GAGCATTGGAAGTTGGGGTA	
MMP-9	F: GCGCCAGCCGACTTATGT	77
	R: AATCCTCTGCCAGCTGTGTGT	
MMP-13	F: TGATGGGCCTTCTGGTCTTCT	59
	R: CCCCGCCAAGGTTTGG	
TIMP-1	F: AAGGGCTACCAGAGCGATCA	80
	R: ATCGAGACCCCAAGGTATTGC	
GAPDH	F: GGCACAGTCAAGGCTGAGAATG	143
	R: ATGGTGGTGAAGACGCCAGTA	

* F: forward; R: reverse; bp: base pairs

### Statistical analysis

Differences in mean values among the experimental groups were analyzed using one-way analysis of variance (ANOVA), followed by Tukey’s HSD and Dunnett’s post-hoc tests with SPSS (version 28.0; SPSS Inc., Chicago, USA). Statistical significance was set as *p* < 0.05.

## Results

### Body weight and gross observations

**Fig. 1:**
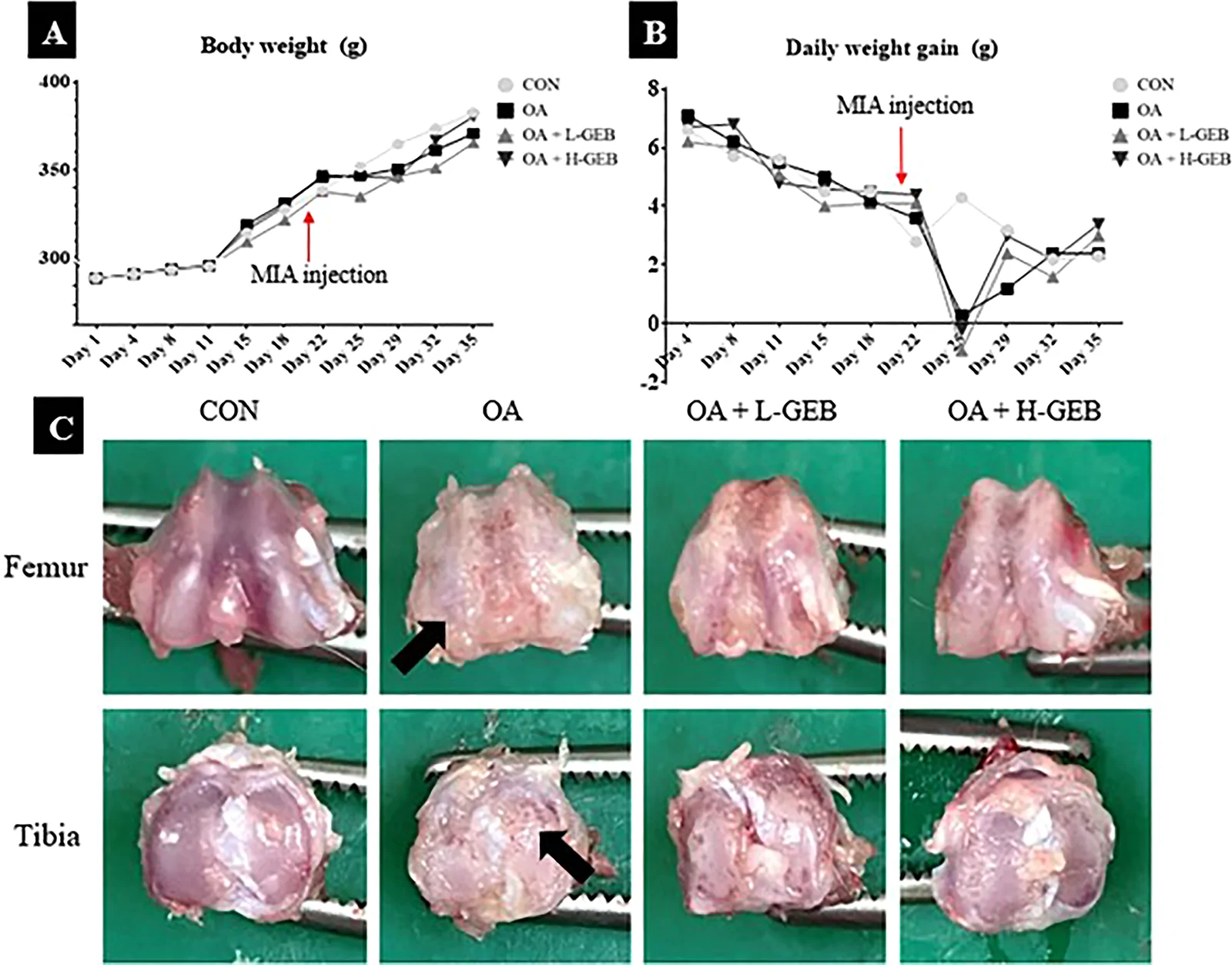
Body weight changes and Gross observation of cartilage surface (**A**) Body weight, (**B**) Daily weight gain, (**C**) Gross observation of cartilage surface: The femoral and tibial surfaces in the OA group showed severe morphological changes, including rough and irregular cartilage surfaces, compared to the smooth cartilage surfaces observed in the CON group. In contrast, the GEB group exhibited fewer surface changes than the OA group

Body weight was monitored twice weekly, and all groups exhibited comparable weight gain. Notably, the OA and GEB-treated groups showed less weight gain compared with the CON group after MIA injection. However, only the OA + H-GEB group recovered to reach a comparable weight level to the CON group (Figs. 1A and B). Histological assessment of the femoral and tibial surfaces revealed that the cartilage of rats in the CON group appeared lustrous and smooth. In contrast, the articular surfaces of rats in the OA group that received MIA were minimally polished and notably rough. However, the articular surfaces of rats in the GEB-treated groups were less rough than those in the OA group (Fig. 1C).

### Histopathological changes in cartilage tissue

**Fig. 2:**
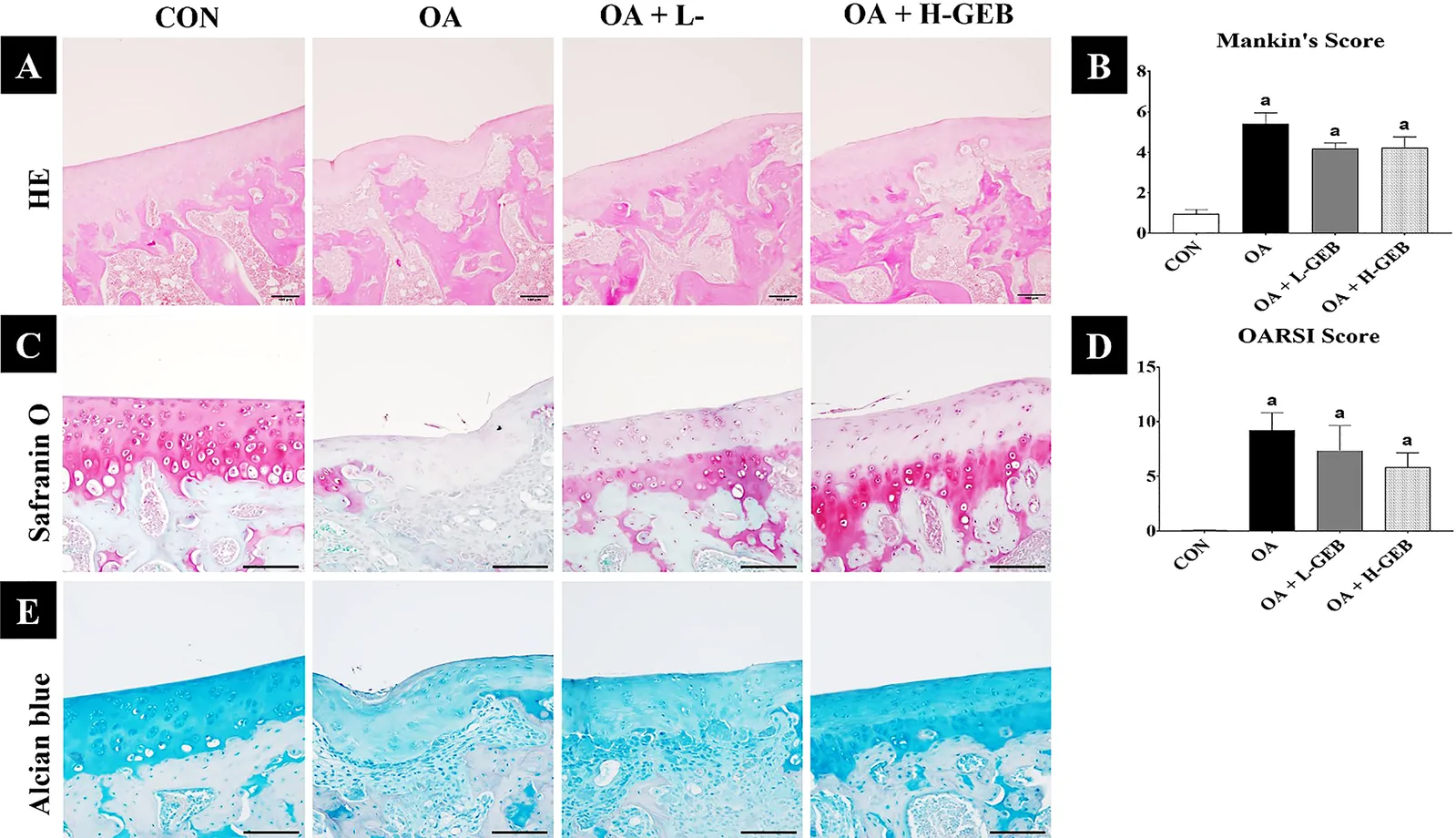
Histopathological assessment in cartilage tissue (**A**) HE staining was performed for histopathological evaluation, and (**B**) the Mankin grading system was applied. (**C**) Safranin O staining was used to evaluate proteoglycan content, and (**D**) the OARSI grading system was applied. (**E**) Alcian blue staining was performed to evaluate glycosaminoglycans. Data are presented as mean ± SE (*n* = 8 per group). (**A**) 100x magnification, (**C** and **E**) 200x magnification; scale bar indicates 100 μm. a = *p* < 0.05 vs. CON

Histopathological examination revealed varying degrees of articular cartilage degeneration across experimental groups. HE staining demonstrated severe cartilage damage characterized by surface irregularities, cartilage thinning, chondrocyte depletion, and increased subchondral fibrosis in the OA group. In contrast, the GEB-treated groups exhibited milder histopathological alterations, with less cartilage thinning and fewer surface irregularities (Fig. 2A). Quantitative assessment of cartilage damage using the Mankin scoring system corroborated the histopathological findings, with the OA group showing significantly higher scores than the CON group. The GEB-treated groups tended to have lower scores than the model group (Fig. 2B). Safranin O and alcian blue staining were performed to further assess the extent of cartilage matrix degradation (Figs. 2C and E). Notably, the histochemical assays revealed that rats in the OA group exhibited significantly reduced proteoglycan content compared with those in the CON group. In contrast, samples in the GEB-treated groups exhibited more intense staining, indicating preserved proteoglycan content. Additionally, the severity of cartilage degradation was further quantified using the Osteoarthritis Research Society International (OARSI) scoring system. Consistent with the histopathological findings, the OA group exhibited significantly higher OARSI scores than the CON group. Although the GEB-treated groups showed higher scores than the CON group, it was lower than that in the OA group (Fig. 2D). The absence of dose-dependent effects observed in the Mankin Score may be attributed to its emphasis on overall histological evaluation, which may obscure subtle changes [46, 47]. In contrast, the OARSI Score focuses on specific and quantifiable cartilage degeneration patterns, allowing more sensitive detection of dose-dependent effects [48].

### IL-6, MMP-13, and caspase-3 expression in cartilage tissue

**Fig. 3:**
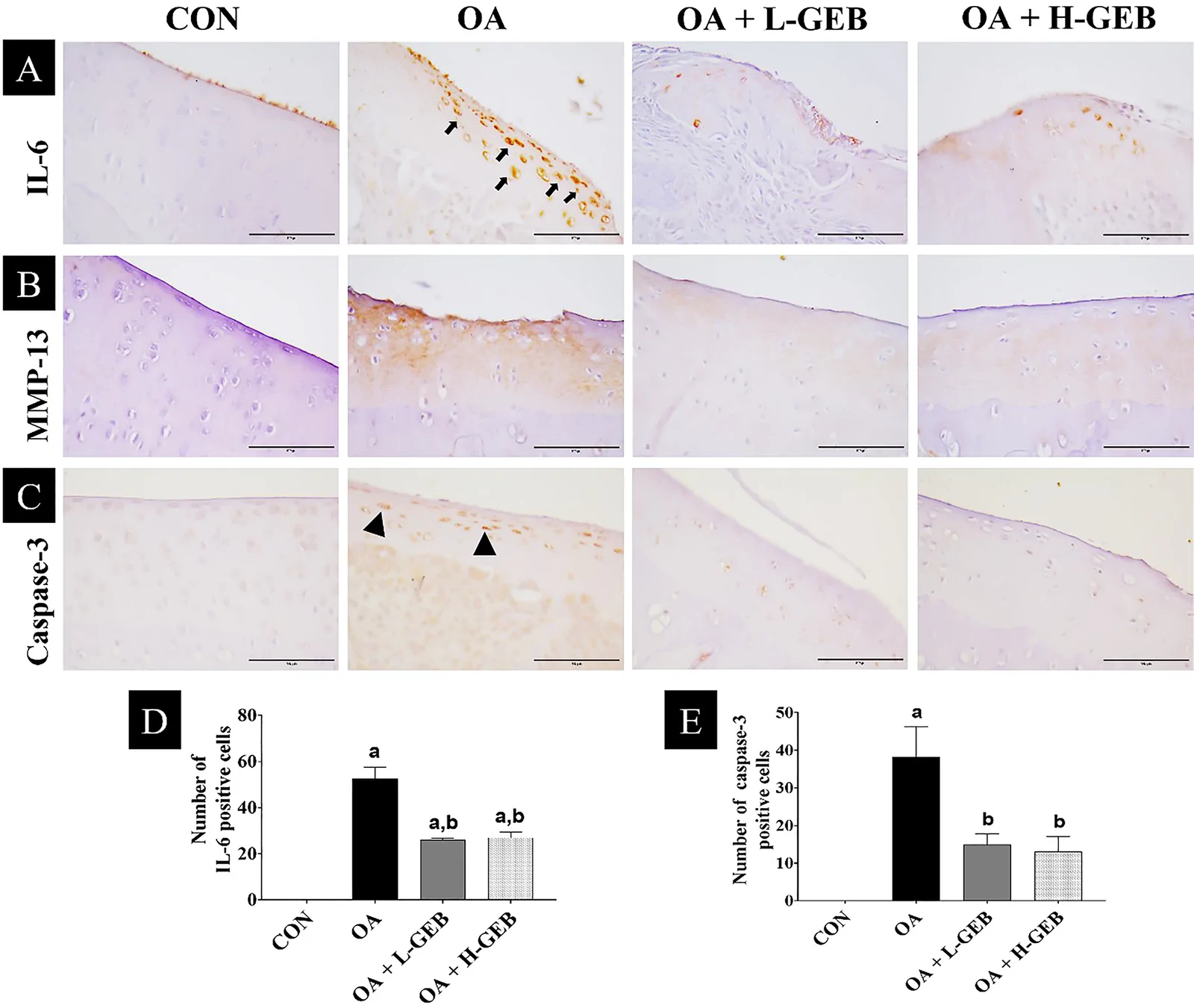
IL-6, MMP-13, and Caspase-3 expression in cartilage tissue of each group The micrographs show the intensity of immunohistochemical staining in each experimental group. (**A**) IL-6, an inflammatory cytokine upregulated by MIA (arrows), showed increased expression in the OA group while its expression was reduced in the GEB-treated groups. (**B**) MMP-13, a cartilage matrix-degrading enzyme, also exhibited increased expression in the OA group and decreased expression in the GEB-treated groups. (**C**) Caspase-3, a crucial enzyme in the process of apoptosis, showed increased expression in the OA group (arrowheads) and decreased expression in the GEB-treated groups compared to the OA group. Data are presented as mean ± SE (*n* = 8 per group). All images were captured at 400x magnification; scale bar indicates 100 μm. a = *p* < 0.05 vs. CON; b = *p* < 0.05 vs. OA

IHC assay was performed to assess the expression of critical inflammatory and degenerative markers in knee joint tissues. Notably, the pro-inflammatory cytokine IL-6 exhibited significantly higher expression in the articular cartilage of rats in the OA group than that in the CON group. However, treatment with GEB extract significantly downregulated IL-6 expression compared with that in the OA group (Fig. 3A). Additionally, we examined the expression of MMPs, which play a pivotal role in extracellular matrix degradation. IHC assay showed that MMP-13 expression was markedly increased in the tibial articular cartilage of rats with MIA-induced OA compared with that in the CON group. However, treatment with GEB extract effectively downregulated MMP-13 staining intensity (Fig. 3B). Additionally, treatment with GEB extract markedly attenuated OA-induced increase in the expression of the apoptosis marker in the cartilage (Fig. 3C).

### TUNEL assay in cartilage tissue

**Fig. 4:**
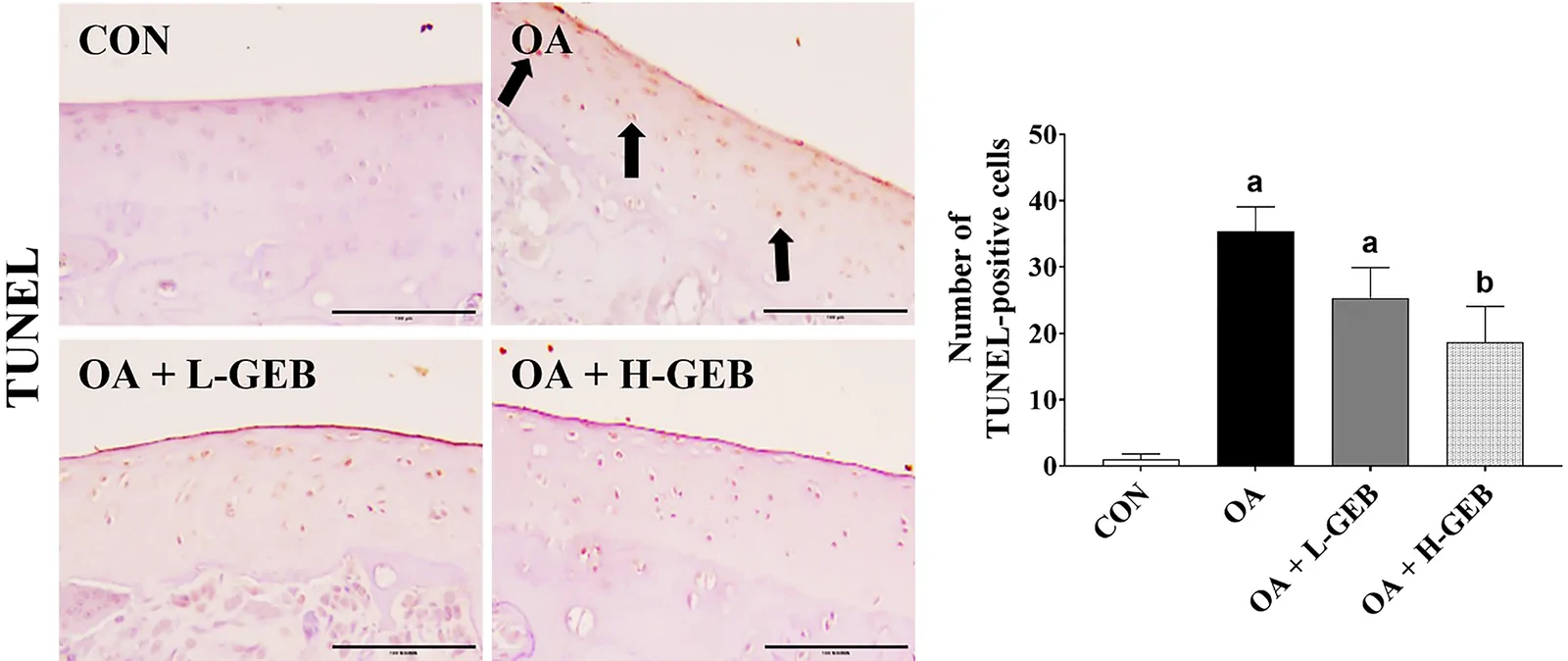
TUNEL assay for detecting apoptotic DNA fragmentation in cartilage tissue TUNEL assay, which detects DNA fragmentation during apoptosis, showed an increase in positive cells in the OA group (arrows), while the GEB-treated groups showed a decrease compared to the OA group. Data are presented as mean ± SE (*n* = 8 per group). All images were captured at 400x magnification; scale bar indicates 100 μm. a = *p* < 0.05 vs. CON; b = *p* < 0.05 vs. OA

TUNEL assay was performed to quantify apoptotic cells in the tissue samples. Consistent with caspase-3 expression level, a marked increase was observed in TUNEL-positive cells in the OA model group compared with that in the CON group. However, GEB treatment significantly reduced the number of TUNEL-positive cells (Fig. 4).

### mRNA expression of nuclear factor kappa B (NF-κB), pro-inflammatory cytokine, and enzymes involved in cartilage degradation

**Fig. 5:**
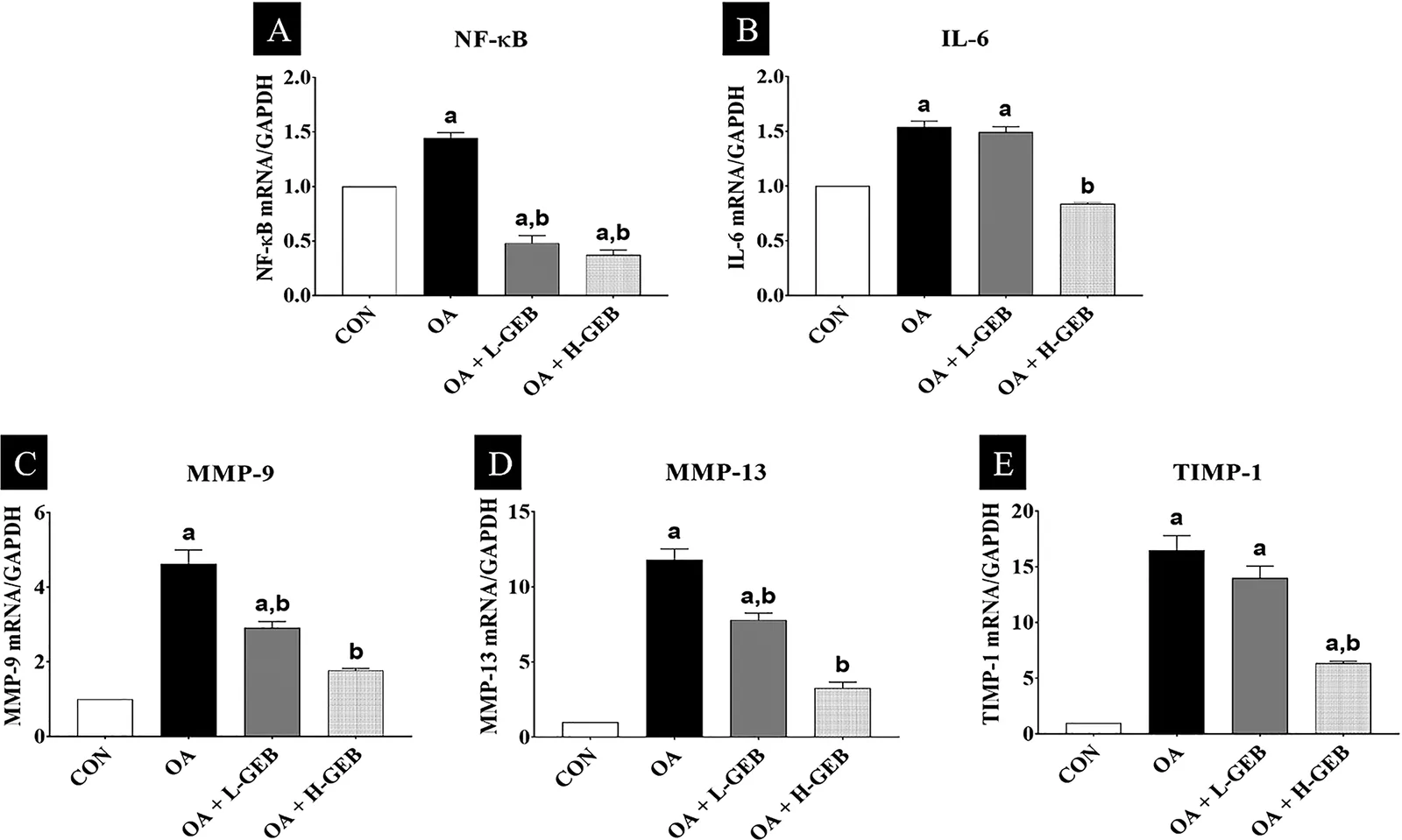
mRNA expression levels of inflammatory and matrix remodeling markers RT-qPCR was performed to evaluate the expression of (**A**) NF-κB, (**B**) IL-6, (**C**) MMP-9, (**D**) MMP-13, and (**E**) TIMP-1. Compared to the CON group, the expression levels of all markers were significantly elevated in the OA group. These markers showed a marked reduction in the OA + L-GEB group, while all markers were significantly decreased to levels comparable to those in the CON group in the OA + H-GEB group. Data are presented as mean ± SE (*n* = 8 per group). a = *p* < 0.05 vs. CON; b = *p* < 0.05 vs. OA

qRT-PCR was performed to examine the expression of critical inflammatory and matrix-degrading factors in the femoral cartilage tissue of the rats. Compared with that in the CON group, a marked increase was observed in the expression of NF-κB, a pivotal regulator of pro-inflammatory genes, in the OA group. However, GEB treatment significantly downregulated NF-κB expression (Fig. 5A). Additionally, treatment with the GEB extract significantly attenuated OA-induced increase in the mRNA levels of the pro-inflammatory cytokine IL-6 (Fig. 5B). Compared with those in the CON group, a significant increase was observed in the mRNA levels of key mediators of cartilage degradation (MMP-9 and MMP-13) and TIMP-1, a regulator of MMP activity, in the OA group. However, treatment with GEB extract effectively modulated this matrix-degrading environment by downregulating MMP-9, MMP-13, and TIMP-1 expression (Figs. 5C, D, and E).

## Discussion

In this study, we investigated the anti-osteoarthritic and anti-inflammatory effects of GEB extract in a rat model of MIA-induced OA. Previous studies have demonstrated the efficacy of GEB extract in modulating diverse inflammatory and immune mechanisms [49–52]. However, its effects and mechanisms in localized inflammatory conditions, such as OA, remain unclear.

OA is characterized by cartilage degeneration primarily driven by chondrocyte apoptosis and subsequent matrix degradation [43, 53, 54]. The MIA-induced OA model used in this study has been widely utilized in prior research and has contributed to our knowledge of the mechanisms underlying cartilage damage [53–56]. MIA administration typically induces joint swelling due to an inflammatory response and weight loss from reduced dietary intake [57, 58]. Consistent with previous findings [59–63], rats in the OA group showed early weight loss and joint swelling compared with those in the CON group. Among the treatment groups, only rats in the H-GEB extract showed weight recovery comparable to that in the CON group. Collectively, these results indicate that OA was locally induced, and body weight changes were used as supportive observations of the general condition of the animals.

Visual observation of articular cartilage during necropsy revealed severe cartilage damage, characterized by a loss of luster and surface roughness, in the OA group. Histopathological assay revealed chondrocyte death, cartilage surface deformation, cartilage layer thinning, subchondral fibrosis, and a marked reduction in proteoglycan levels in the OA model group compared with those in the CON group. Consistent with prior reports [64–66], our findings reflect characteristic pathological alterations associated with MIA-induced cartilage destruction. Compared with those in the OA group, rats in the GEB-treated groups exhibited fewer lesions and less severe cartilage matrix damage, which was confirmed by Mankin and OARSI scores [67–69]. Collectively, these results suggest that GEB extract mitigates MIA-induced cartilage destruction, demonstrating anti-osteoarthritic activity.

OA is primarily driven by MMPs and inflammatory mediators [70, 71]. Our study showed that the mRNA levels of key components, including MMPs and TIMPs, were significantly upregulated in the cartilage of rats with MIA-induced OA compared with that in the CON group. MMPs play a pivotal role in cartilage matrix degradation, with MMP-13 being particularly effective in degrading type II collagen and TIMPs inhibiting their activity [72–75]. Research findings indicate that suppressing proteolytic activity and expression of specific MMPs may mitigate the progression of articular cartilage degradation [76, 77]. The increased TIMP-1 mRNA expression in the OA group suggests negative feedback regulation of MMPs [78]. GEB extract downregulated the expression of both MMPs and TIMP-1, indicating effective inhibition of cartilage matrix degradation. Notably, this balance between MMPs and TIMPs represents a crucial mechanism for cartilage protection [79, 80]. Overall, these results suggest that GEB extract exerts chondroprotective effects by preserving this equilibrium, thereby inhibiting articular cartilage matrix degradation.

Inflammatory cytokines play a crucial role in the development and progression of OA [81, 82]. In MIA-induced OA models, NF-κB upregulation in cartilage tissue promotes IL-6 expression and stimulates the release of catabolic enzymes, such as MMPs, further exacerbating the disease [83, 84]. Therefore, modulating this pathway has been identified as a potential strategy for inhibiting OA progression [83]. qRT-PCR showed that there was a significant upregulation in NF-κB and IL-6 mRNA levels in the OA group. However, treatment with GEB extract attenuated the expression of these inflammatory factors, suggesting that GEB extract protects against chondrocyte degradation by suppressing the activation of pro-inflammatory factors.

Cell death pathways activated by various stress stimuli, including inflammation, promote chondrocyte apoptosis and contribute cartilage damage in OA [85–87]. In this study, cleaved caspase-3 and TUNEL staining were used as supportive indicators of apoptosis-associated chondrocyte damage, rather than to determine whether apoptosis was the dominant cell death mechanism in the inflammatory OA context. Therefore, we examined the expression cleaved caspase-3 (apoptosis marker) and DNA damage in cartilage tissue using IHC and TUNEL assays, respectively [88, 89]. Treatment with the GEB extract significantly attenuated OA-induced upregulation in caspase-3 expression and TUNEL-positive cells in the cartilage of rats. Similarly, previous studies have shown that specific natural substances exert chondroprotective effects through the inhibition of cell death [90, 91].

Although not statistically significant, histopathological analyses using HE and Safranin O staining indicated a decreasing trend in Mankin and OARSI scores in the GEB-treated groups compared with those in the OA group. Overall, these findings suggest that the marginal improvement in Mankin and OARSI scores may be attributable to the prolonged timeframe required for structural restoration of cartilage, as assessed through histopathological scoring, in contrast to the relatively rapid molecular alterations in IL-6, MMP-13, cleaved caspase-3, and NF-κB observed through RT-qPCR, IHC, and TUNEL assays. However, the histopathological findings revealed statistical significance in the linear trend analysis (*p* = 0.0332), supporting the preventive effect of GEB extract. GEB extract modulates key inflammatory pathways (NF-κB and IL-6), suppresses apoptosis (evidenced by decreased cleaved caspase-3 expression and TUNEL-positive cells), and maintains the equilibrium of MMPs and TIMP activity, highlighting its potential as a promising preventive candidate for OA.

Our study highlights the therapeutic potential of GEB extract in mitigating OA through multiple mechanisms, including preserving cartilage integrity. Although the structural restoration observed through histopathological analysis was not statistically significant, the trend analysis (*p* = 0.0332) supports the potential of GEB extract as a preventive candidate. Therefore, longer treatment durations may be required for significant structural recovery, consistent with previous findings that long-term treatment with natural compounds is necessary to achieve visible histological improvements.

At the molecular level, the ability of GEB extract to suppress NF-κB and IL-6 expression suggests its role in modulating key inflammatory pathways, emphasizing the importance of targeting these pathways in OA management. GEB extract-mediated regulation of MMPs and TIMPs further highlights its role in maintaining cartilage matrix homeostasis. GEB extract appears to effectively inhibit cartilage degradation by balancing proteolytic activity. Additionally, the observed decrease in cleaved caspase-3 expression and TUNEL-positive cells indicates that GEB extract inhibits chondrocyte apoptosis. Collectively, these findings suggest that GEB extract not only alleviates inflammation but also preserves the structural and functional integrity of cartilage, making it promising preventive candidate for OA.

Notably, this study underscores the importance of GEB extract in addressing the complex pathophysiology of OA. Although the findings provide robust molecular evidence of the efficacy of GEB extract, future studies should focus on long-term applications and explore the potential of GEB extract in combination with other therapies to maximize its protective impact.

Conclusively, this study demonstrates the potential of GEB extract to modulate key inflammatory pathways in rat model of OA, highlighting its role as a promising natural preventive candidate. GEB extract demonstrates anti-inflammatory, anti-osteoarthritis, and immunomodulatory effects. The extract contains various bioactive compounds, such as GAS and HBA, and their interactions may contribute to the complex effects observed in this study. However, the precise molecular mechanisms of action and potential interactions of these active constituents remain unclear. In addition, the absence of a positive control, such as indomethacin, is a limitation of this study. Another limitation is that the anti-OA effects of GEB extract were evaluated only in the MIA-induced OA model. Although the MIA-induced model is widely used as a rodent OA model [92, 93], it does not fully reflect the heterogeneous pathophysiology of human OA. Further studies using additional OA or arthritis-related models, such as surgically induced OA, CIA, or CAIA models [94, 95], are warranted to elucidate these mechanisms, assess the long-term safety of GEB extract, and strengthen the translational relevance of these findings.

## Conclusions

**Fig. 6:**
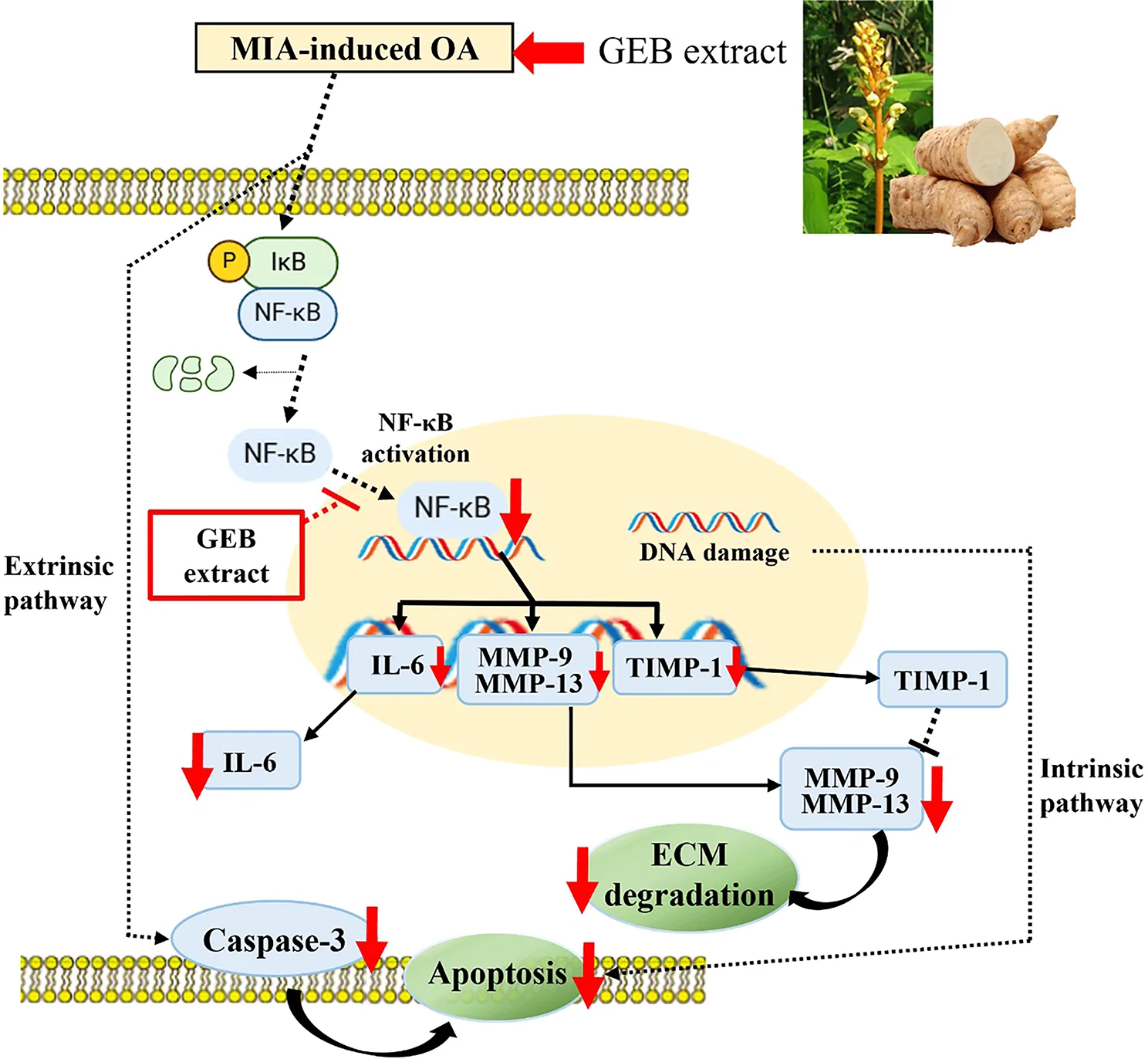
The suggested mechanisms on the effects of *Gastrodia elata* Blume extract

This study demonstrated the anti-inflammatory and immunomodulatory effects of GEB extract in a rat model of MIA-induced OA, highlighting its potential as a natural preventive natural candidate for mitigating articular inflammation. GEB extract effectively suppresses cartilage degradation by modulating MMPs and TIMPs, suppressing IL-6, and inhibiting chondrocyte apoptosis, thereby preserving cartilage integrity (Fig. 6). Although these findings provide valuable insights, further studies are needed to clarify the precise molecular mechanisms involved and assess the long-term safety of GEB extract. Future research should also explore its efficacy in other inflammatory disease models to establish broader preventive applicability.

## Supplementary Information

Below is the link to the electronic supplementary material.

Supplementary Material 1: Experimental design and grouping for MIA-induced OA model in rats. Rats were divided into four groups (n = 8 per group): CON, OA, OA + L-GEB, and OA + H-GEB. OA was induced via a single intra-articular injection of monosodium iodoacetate (MIA, 3 mg/50 µL saline). GEB extract or distilled water (DW) was orally administered (p.o.) daily for 5 weeks. Rats were sacrificed 24 h after the last treatment

## Data Availability

No datasets were generated or analysed during the current study.

## Abbreviations

GEB: *Gastrodia elata* Blume
GAS: Gastrodin
HBA: 4-Hydroxybenzyl Alcohol
OA: Osteoarthritis
MIA: Monosodium iodoacetate
GAPDH: Glyceraldehyde-3-phosphate dehydrogenase
DAMPs: Damage-associated molecular patterns
PRRs: Pattern recognition receptors
MMP: Matrix metalloproteinase
TIMP: Tissue inhibitor of metalloproteinase
NSAID: Non-steroidal anti-inflammatory drug
DW: Distilled water
QC: Quality control
IHC: Immunohistochemical
IL-6: Interleukin-6
TUNEL: Terminal deoxynucleotidyl transferase dUTP nick end labeling
SD: Spraque-Dawley
RT-qPCR: Quantitative reverse transcription polymerase chain reaction
NBF: Neutral buffered formalin
GAG: Glycosaminoglycan
HCl: Hydrogen chloride
PBS: Phosphate-buffered saline
DAB: 3,3-diaminobenzidine tetrahydrochloride
ANOVA: one-way analysis of variance
NF-κB: Nuclear factor kappa B

## References

[1] Cheng L, Wang H, Ma K, Deng Y, Li M, Ma J. A novel alcohol steamed preparation from Gastrodia elata Blume: Pharmacological assessment of a functional food. Front Pharmacol. 2023;14:1092693. 10.3389/fphar.2023.1092693 PMC10073492 37033659

[2] Gong MQ, Lai FF, Chen JZ, Li XH, Chen YJ, He Y. Traditional uses, phytochemistry, pharmacology, applications, and quality control of Gastrodia elata Blume: A comprehensive review. J Ethnopharmacol. 2024;319(Pt 1):117128. 10.1016/j.jep.2023.117128 37689324

[3] Lu C, Qu S, Zhong Z, Luo H, Lei SS, Zhong HJ, et al. The effects of bioactive components from the rhizome of gastrodia elata blume (Tianma) on the characteristics of Parkinson’s disease. Front Pharmacol. 2022;13:963327. 10.3389/fphar.2022.963327 PMC9748092 36532787

[4] Yang L, Qin SH, Zi CT. Research progress of Gastrodia elata Blume polysaccharides: a review of chemical structures and biological activities. Front Chem. 2024;12:1395222. 10.3389/fchem.2024.1395222 PMC11250123 39015542

[5] Zhang Y, Huang H, Yao C, Sun X, He Q, Choudharyc MI, et al. Fresh Gastrodia elata Blume alleviates simulated weightlessness-induced cognitive impairment by regulating inflammatory and apoptosis-related pathways. Front Pharmacol. 2023;14:1173920. 10.3389/fphar.2023.1173920 PMC10188943 37205911

[6] Zhou HB, Lu SZ, Yu ZS, Zhang JL, Mei ZN. Mechanisms for the biological activity of Gastrodia elata Blume and its constituents: A comprehensive review on sedative-hypnotic, and antidepressant properties. Phytomedicine. 2024;123:155251. 10.1016/j.phymed.2023.155251 38056151

[7] Zhu H, Liu C, Hou J, Long H, Wang B, Guo D, et al. Gastrodia elata Blume Polysaccharides: A Review of Their Acquisition, Analysis, Modification, and Pharmacological Activities. Molecules. 2019;24(13):2436. 10.3390/molecules24132436 PMC6651794 31269719

[8] Chen X-Y, He Y, Chen J-Y. Pharmacological effects and mechanisms of Gastrodia elata and its active ingredients in the treatment of cardiovascular diseases. Tradit Med Res. 2023;8(9):52.

[9] He X, Yang Y, Yuan X, Sun Y, Li Y. Chemical composition and anticonvulsant activities of herb pair of Gastrodia elata Blume–Acorus tatarinowii Schott decoction on experimentally induced seizures in mice. Metab Brain Dis. 2023;38(6):1877–93. 10.1007/s11011-023-01211-9 37043151

[10] Hsieh CL, Chiang SY, Cheng KS, Lin YH, Tang NY, Lee CJ, et al. Anticonvulsive and free radical scavenging activities of Gastrodia elata Bl. in kainic acid-treated rats. Am J Chin Med. 2001;29(2):331–41. 10.1142/S0192415X01000356 11527075

[11] Lee YS, Park YR, Lee HB, Park HJ, Lee HE, Kim GA, et al. The preventive effect of Gastrodia elata Blume extract on vancomycin-induced acute kidney injury in rats. Lab Anim Res. 2024;40(1):14. 10.1186/s42826-024-00200-y PMC11000351 38589968

[12] Seok PR, Kim JH, Kwon HR, Heo JS, Choi JR, Shin JH. Protective effects of Gastrodia elata Blume on acetaminophen-induced liver and kidney toxicity in rats. Food Sci Biotechnol. 2018;27(5):1445–54. 10.1007/s10068-018-0374-5 PMC6170282 30319855

[13] Wu YN, Wen SH, Zhang W, Yu SS, Yang K, Liu D, et al. Gastrodia elata BI.:A Comprehensive Review of Its Traditional Use, Botany, Phytochemistry, Pharmacology, and Pharmacokinetics. Evid Based Complement Alternat Med. 2023;2023:5606021. 10.1155/2023/5606021 PMC10129437 37114145

[14] Wang Z, Zuo C, Chen M, Song J, Tu K, Lan W, et al. A Novel Variable Selection Method Based on Ordered Predictors Selection and Successive Projections Algorithm for Predicting Gastrodin Content in Fresh Gastrodia elata Using Fourier Transform Near-Infrared Spectroscopy and Chemometrics. Foods. 2023;12(24):4435. 10.3390/foods12244435 PMC10743185 38137239

[15] Huang H, Zhang Y, Yao C, He Q, Chen F, Yu H, et al. The effects of fresh Gastrodia elata Blume on the cognitive deficits induced by chronic restraint stress. Front Pharmacol. 2022;13:890330. 10.3389/fphar.2022.890330 PMC9464977 36105220

[16] Kim NH, Xin MJ, Cha JY, Ji SJ, Kwon SU, Jee HK, et al. Antitumor and Immunomodulatory Effect of Gastrodia elata on Colon Cancer In Vitro and In Vivo. Am J Chin Med. 2017;45(2):319–35. 10.1142/S0192415X17500203 28231745

[17] Heo YH, Choi NH, Seo YK, Jang JY, Lee JH, Kim JK, et al. Anti-inflammatory Effects of Gastrodia elata Extract in Lipopolysaccharide-stimulated BV-2 Microglia Cell. J Physiol Pathol Korean Med. 2012;26(5):738–44.

[18] Guan VX, Mobasheri A, Probst YC. A systematic review of osteoarthritis prevention and management with dietary phytochemicals from foods. Maturitas. 2019;122:35–43. 10.1016/j.maturitas.2019.01.005 30797528

[19] Lakshmanan DK, Ravichandran G, Elangovan A, Jeyapaul P, Murugesan S, Thilagar S. Cissus quadrangularis (veldt grape) attenuates disease progression and anatomical changes in mono sodium iodoacetate (MIA)-induced knee osteoarthritis in the rat model. Food Funct. 2020;11(9):7842–55. 10.1039/d0fo00992j 32812575

[20] An JS, Tsuji K, Onuma H, Araya N, Isono M, Hoshino T, et al. Inhibition of fibrotic changes in infrapatellar fat pad alleviates persistent pain and articular cartilage degeneration in monoiodoacetic acid-induced rat arthritis model. Osteoarthritis Cartilage. 2021;29(3):380–8. 10.1016/j.joca.2020.12.014 33388431

[21] Bhore P, Shinde S. Effect of multi-component exercises program on pain-related gait adaptations among individuals with osteoarthritis of the knee joint. J Educ Health Promot. 2023;12:138. 10.4103/jehp.jehp_1628_22 PMC10312405 37397115

[22] D’Amico D, Olmer M, Fouassier AM, Valdes P, Andreux PA, Rinsch C, et al. Urolithin A improves mitochondrial health, reduces cartilage degeneration, and alleviates pain in osteoarthritis. Aging Cell. 2022;21(8):e13662. 10.1111/acel.13662 PMC9381911 35778837

[23] Li K, Ji X, Seeley R, Lee WC, Shi Y, Song F, et al. Impaired glucose metabolism underlies articular cartilage degeneration in osteoarthritis. FASEB J. 2022;36(6):e22377. 10.1096/fj.202200485R 35608871

[24] Yue L, Berman J. What Is Osteoarthritis? JAMA. 2022;327(13):1300. 10.1001/jama.2022.1980 35380583

[25] Radin EL, Paul IL, Rose RM. Role of mechanical factors in pathogenesis of primary osteoarthritis. Lancet. 1972;1(7749):519–22. 10.1016/s0140-6736(72)90179-1 4110024

[26] Blom AB, van Lent PL, Libregts S, Holthuysen AE, van der Kraan PM, van Rooijen N, et al. Crucial role of macrophages in matrix metalloproteinase-mediated cartilage destruction during experimental osteoarthritis: involvement of matrix metalloproteinase 3. Arthritis Rheum. 2007;56(1):147–57. 10.1002/art.22337 17195217

[27] de Lange-Brokaar BJ, Kloppenburg M, Andersen SN, Dorjee AL, Yusuf E, Herb-van Toorn L, et al. Characterization of synovial mast cells in knee osteoarthritis: association with clinical parameters. Osteoarthritis Cartilage. 2016;24(4):664–71. 10.1016/j.joca.2015.11.011 26671522

[28] Kraus VB, McDaniel G, Huebner JL, Stabler TV, Pieper CF, Shipes SW, et al. Direct in vivo evidence of activated macrophages in human osteoarthritis. Osteoarthritis Cartilage. 2016;24(9):1613–21. 10.1016/j.joca.2016.04.010 PMC4992586 27084348

[29] Li YS, Luo W, Zhu SA, Lei GH. T Cells in Osteoarthritis: Alterations and Beyond. Front Immunol. 2017;8:356. 10.3389/fimmu.2017.00356 PMC5371609 28424692

[30] Kapoor M, Martel-Pelletier J, Lajeunesse D, Pelletier JP, Fahmi H. Role of proinflammatory cytokines in the pathophysiology of osteoarthritis. Nat Rev Rheumatol. 2011;7(1):33–42. 10.1038/nrrheum.2010.196 21119608

[31] Philp AM, Davis ET, Jones SW. Developing anti-inflammatory therapeutics for patients with osteoarthritis. Rheumatology (Oxford). 2017;56(6):869–81. 10.1093/rheumatology/kew278 27498352

[32] Chen C, Zhou H, Yin Y, Hu H, Jiang B, Zhang K, et al. Rotator cuff muscle degeneration in a mouse model of glenohumeral osteoarthritis induced by monoiodoacetic acid. J Shoulder Elb Surg. 2023;32(3):500–11. 10.1016/j.jse.2022.10.026 36442828

[33] Choi DJ, Choi SI, Choi BR, Lee YS, Lee DY, Kim GS. Cartilage protective and anti-analgesic effects of ALM16 on monosodium iodoacetate induced osteoarthritis in rats. BMC Complement Altern Med. 2019;19(1):325. 10.1186/s12906-019-2746-7 PMC6873692 31752825

[34] Jung JI, Lee HS, Jeon YE, Kim SM, Hong SH, Moon JM, et al. Anti-inflammatory activity of palmitoylethanolamide ameliorates osteoarthritis induced by monosodium iodoacetate in Sprague-Dawley rats. Inflammopharmacology. 2021;29(5):1475–86. 10.1007/s10787-021-00870-3 PMC8514352 34468900

[35] Orlowsky EW, Kraus VB. The role of innate immunity in osteoarthritis: when our first line of defense goes on the offensive. J Rheumatol. 2015;42(3):363–71. 10.3899/jrheum.140382 PMC4465583 25593231

[36] Amir M, Czaja MJ. Inflammasome-mediated inflammation and fibrosis: It is more than just the IL-1beta. Hepatology. 2018;67(2):479–81. 10.1002/hep.29491 PMC5832548 28862748

[37] Burrage PS, Mix KS, Brinckerhoff CE. Matrix metalloproteinases: role in arthritis. Front Biosci. 2006;11:529–43. 10.2741/1817 16146751

[38] Ferreira-Gomes J, Garcia MM, Nascimento D, Almeida L, Quesada E, Castro-Lopes JM, et al. TLR4 Antagonism Reduces Movement-Induced Nociception and ATF-3 Expression in Experimental Osteoarthritis. J Pain Res. 2021;14:2615–27. 10.2147/JPR.S317877 PMC8403032 34466029

[39] Pascual M, Calvo-Rodriguez M, Nunez L, Villalobos C, Urena J, Guerri C. Toll-like receptors in neuroinflammation, neurodegeneration, and alcohol-induced brain damage. IUBMB Life. 2021;73(7):900–15. 10.1002/iub.2510 34033211

[40] Reynolds JJ, Hembry RM, Meikle MC. Connective tissue degradation in health and periodontal disease and the roles of matrix metalloproteinases and their natural inhibitors. Adv Dent Res. 1994;8(2):312–9. 10.1177/08959374940080022701 7865092

[41] Robert S, Gicquel T, Bodin A, Lagente V, Boichot E. Characterization of the MMP/TIMP Imbalance and Collagen Production Induced by IL-1beta or TNF-alpha Release from Human Hepatic Stellate Cells. PLoS ONE. 2016;11(4):e0153118. 10.1371/journal.pone.0153118 PMC4821480 27046197

[42] Wang X, Antony V, Wang Y, Wu G, Liang G. Pattern recognition receptor-mediated inflammation in diabetic vascular complications. Med Res Rev. 2020;40(6):2466–84. 10.1002/med.21711 32648967

[43] Oh DK, Na HS, Jhun JY, Lee JS, Um IG, Lee SY, et al. Bifidobacterium longum BORI inhibits pain behavior and chondrocyte death, and attenuates osteoarthritis progression. PLoS ONE. 2023;18(6):e0286456. 10.1371/journal.pone.0286456 PMC10289443 37352198

[44] Lu K-H, Ou G-L, Chang H-P, Chen W-C, Liu S-H, Sheen L-Y. Safety evaluation of water extract of Gastrodia elata Blume: Genotoxicity and 28-day oral toxicity studies. Regul Toxicol Pharmacol. 2020;114:104657. 10.1016/j.yrtph.2020.104657 32278877

[45] Yu B, Zuo Z, Kong W. Experimental studies of the toxicity and safety of Gastrodina elata powder tablets. China Mod Med. 2014;21(21):6–10.

[46] Moody HR, Heard BJ, Frank CB, Shrive NG, Oloyede AO. Investigating the potential value of individual parameters of histological grading systems in a sheep model of cartilage damage: the Modified Mankin method. J Anat. 2012;221(1):47–54. 10.1111/j.1469-7580.2012.01513.x PMC3390533 22591160

[47] Ostergaard K, Petersen J, Andersen CB, Bendtzen K, Salter DM. Histologic/histochemical grading system for osteoarthritic articular cartilage: reproducibility and validity. Arthritis Rheum. 1997;40(10):1766–71. 10.1002/art.1780401007 9336409

[48] Ma L, Liu Y, Zhao X, Li P, Jin Q. Rapamycin attenuates articular cartilage degeneration by inhibiting beta-catenin in a murine model of osteoarthritis. Connect Tissue Res. 2019;60(5):452–62. 10.1080/03008207.2019.1583223 30782019

[49] Huang HS, Lin YE, Panyod S, Chen RA, Lin YC, Chai LMX, et al. Anti-depressive-like and cognitive impairment alleviation effects of Gastrodia elata Blume water extract is related to gut microbiome remodeling in ApoE(-/-) mice exposed to unpredictable chronic mild stress. J Ethnopharmacol. 2023;302(Pt B):115872. 10.1016/j.jep.2022.115872 36343797

[50] Shi X, Yu X, Yang L, Duan X. Ethyl acetate extract of Gastrodia elata protects Caenorhabditis elegans from oxidative stress and amyloid beta peptide toxicity. Exp Ther Med. 2023;26(2):405. 10.3892/etm.2023.12104 PMC10375435 37522064

[51] Wang D, Wang JX, Yan C, Liu Y, Liu H, Li D, et al. Gastrodia elata Blume extract improves high-fat diet-induced type 2 diabetes by regulating gut microbiota and bile acid profile. Front Microbiol. 2022;13:1091712. 10.3389/fmicb.2022.1091712 PMC9756436 36532435

[52] Zhang T, Huang S, Qiu J, Wu X, Yuan H, Park S. Beneficial Effect of Gastrodia elata Blume and Poria cocos Wolf Administration on Acute UVB Irradiation by Alleviating Inflammation through Promoting the Gut-Skin Axis. Int J Mol Sci. 2022;23(18):10833. 10.3390/ijms231810833 PMC9504230 36142744

[53] Li Z, Huang Z, Zhang H, Lu J, Tian Y, Wei Y, et al. P2X7 Receptor Induces Pyroptotic Inflammation and Cartilage Degradation in Osteoarthritis via NF-kappaB/NLRP3 Crosstalk. Oxid Med Cell Longev. 2021;2021:8868361. 10.1155/2021/8868361 PMC7834826 33532039

[54] Yan L, Zhou L, Xie D, Du W, Chen F, Yuan Q, et al. Chondroprotective effects of platelet lysate towards monoiodoacetate-induced arthritis by suppression of TNF-α-induced activation of NF-ĸB pathway in chondrocytes. Aging. 2019;11(9):2797. 10.18632/aging.101952 PMC6535074 31089001

[55] Chiu PR, Hu YC, Huang TC, Hsieh BS, Yeh JP, Cheng HL, et al. Vitamin C Protects Chondrocytes against Monosodium Iodoacetate-Induced Osteoarthritis by Multiple Pathways. Int J Mol Sci. 2016;18(1):38. 10.3390/ijms18010038 PMC5297673 28035982

[56] Siracusa R, Impellizzeri D, Cordaro M, Peritore AF, Gugliandolo E, D’amico R, et al. The protective effect of new carnosine-hyaluronic acid conjugate on the inflammation and cartilage degradation in the experimental model of osteoarthritis. Appl Sci. 2020;10(4):1324.

[57] Goldring MB, Otero M. Inflammation in osteoarthritis. Curr Opin Rheumatol. 2011;23(5):471–8. 10.1097/BOR.0b013e328349c2b1 PMC3937875 21788902

[58] Salucci S, Falcieri E, Battistelli M. Chondrocyte death involvement in osteoarthritis. Cell Tissue Res. 2022;389(2):159–70. 10.1007/s00441-022-03639-4 PMC9287242 35614364

[59] El-Seweidy MM, Sousou I, Elswefy SE, Mashhour MM. Omega 3-fatty acids, atorvastatin as modulators for inflammatory pattern versus diclofenac in osteoarthritis induced in experimental rats. Afr J Pharm Pharmacol. 2016;10(22):472–9.

[60] Gorkava I, Maliuk M. Changes in the number of white blood cells and non-specific markers of inflammation in the body of rabbits in experimental osteoarthritis of the knee joint. Ukr J Vet Sci. 2022;13(3):9–16.

[61] Hahm S-C, Lee G-E, Kim E-H, Kim J, Lee T, Lee W. The effects of pre-emptive low-dose X-ray irradiation on MIA induced inflammatory pain in rats. J Korean Phys Soc. 2013;63:269–75.

[62] Lee YM, Son E, Kim SH, Kim DS. Protective Effects of Glycine soja Leaf and Stem Extract against Chondrocyte Inflammation and Osteoarthritis. Int J Mol Sci. 2023;24(5):4829. 10.3390/ijms24054829 PMC10002952 36902256

[63] Recinella L, Micheli L, Chiavaroli A, Libero ML, Orlando G, Menghini L, et al. Anti-Inflammatory Effects Induced by a Polyphenolic Granular Complex from Olive (Olea europaea, Mainly Cultivar coratina): Results from In Vivo and Ex Vivo Studies in a Model of Inflammation and MIA-Induced Osteoarthritis. Nutrients. 2022;14(7):1487. 10.3390/nu14071487 PMC9002755 35406100

[64] Bao Z, Chen M, Li C, Shan Q, Wang Y, Yang W. Monosodium iodoacetate-induced subchondral bone microstructure and inflammatory changes in an animal model of osteoarthritis. Open Life Sci. 2022;17(1):781–93. 10.1515/biol-2022-0079 PMC9287849 35903181

[65] Xie L, Lin AS, Kundu K, Levenston ME, Murthy N, Guldberg RE. Quantitative imaging of cartilage and bone morphology, reactive oxygen species, and vascularization in a rodent model of osteoarthritis. Arthritis Rheum. 2012;64(6):1899–908. 10.1002/art.34370 PMC3366030 22231023

[66] Yang D-H, Woo C-H, Kim J-M, An H-D. Effects of Danggwisayeok-tang (Dangguisinitang) on MIA-induced osteoarthritis rats. J Korean Med Rehabilitation. 2015;25(2):37–50.

[67] Hamdalla HM, Ahmed RR, Galaly SR, Ahmed OM, Naguib IA, Alghamdi BS, et al. Assessment of the Efficacy of Bone Marrow-Derived Mesenchymal Stem Cells against a Monoiodoacetate-Induced Osteoarthritis Model in Wistar Rats. Stem Cells Int. 2022;2022:1900403. 10.1155/2022/1900403 PMC9398859 36017131

[68] Moskowitz RW. Osteoarthritis cartilage histopathology: grading and staging. Osteoarthritis Cartilage. 2006;14(1):1–2. 10.1016/j.joca.2005.08.015 16242362

[69] Pearson RG, Kurien T, Shu KS, Scammell BE. Histopathology grading systems for characterisation of human knee osteoarthritis–reproducibility, variability, reliability, correlation, and validity. Osteoarthritis Cartilage. 2011;19(3):324–31. 10.1016/j.joca.2010.12.005 21172446

[70] Afifah E, Mozef T, Sandra F, Arumwardana S, Rihibiha DD, Nufus H, et al. Induction of matrix metalloproteinases in chondrocytes by interleukin IL-1β as an osteoarthritis model. J Math Fund Sci. 2019;51(2):103–11.

[71] Bryk M, Chwastek J, Mlost J, Kostrzewa M, Starowicz K. Sodium Monoiodoacetate Dose-Dependent Changes in Matrix Metalloproteinases and Inflammatory Components as Prognostic Factors for the Progression of Osteoarthritis. Front Pharmacol. 2021;12:643605. 10.3389/fphar.2021.643605 PMC8113822 33995052

[72] Dharmayuda CGO, Kawiyana IKS, Bakta IM, Astawa INM, Astawa P, Manuaba IBP, et al. Administration of mesenchymal stem cell derivate secretome causes lower levels of Matrix Metalloproteinase-13, Aggrecanase-2, and Interleukin-1β than hyaluronic acid in rabbit knee osteoarthritis. Bali Med J. 2023;12(1):899–903.

[73] Jungel A, Ospelt C, Lesch M, Thiel M, Sunyer T, Schorr O, et al. Effect of the oral application of a highly selective MMP-13 inhibitor in three different animal models of rheumatoid arthritis. Ann Rheum Dis. 2010;69(5):898–902. 10.1136/ard.2008.106021 PMC2925150 19497915

[74] Qu H, Li J, Wu LD, Chen WP. Trichostatin A increases the TIMP-1/MMP ratio to protect against osteoarthritis in an animal model of the disease. Mol Med Rep. 2016;14(3):2423–30. 10.3892/mmr.2016.5523 PMC4991690 27431944

[75] Visse R, Nagase H. Matrix metalloproteinases and tissue inhibitors of metalloproteinases: structure, function, and biochemistry. Circ Res. 2003;92(8):827–39. 10.1161/01.RES.0000070112.80711.3D 12730128

[76] Li NG, Shi ZH, Tang YP, Wang ZJ, Song SL, Qian LH, et al. New hope for the treatment of osteoarthritis through selective inhibition of MMP-13. Curr Med Chem. 2011;18(7):977–1001. 10.2174/092986711794940905 21254976

[77] Murphy G, Knauper V, Atkinson S, Butler G, English W, Hutton M, et al. Matrix metalloproteinases in arthritic disease. Arthritis Res. 2002;4(Suppl 3Suppl 3):S39–49. 10.1186/ar572 PMC3240148 12110122

[78] Yang Y, Wang Y, Kong Y, Zhang X, Bai L. The effects of different frequency treadmill exercise on lipoxin A4 and articular cartilage degeneration in an experimental model of monosodium iodoacetate-induced osteoarthritis in rats. PLoS ONE. 2017;12(6):e0179162. 10.1371/journal.pone.0179162 PMC5464632 28594958

[79] Chen GY, Chen JQ, Liu XY, Xu Y, Luo J, Wang YF, et al. Total Flavonoids of Rhizoma Drynariae Restore the MMP/TIMP Balance in Models of Osteoarthritis by Inhibiting the Activation of the NF-kappaB and PI3K/AKT Pathways. Evid Based Complement Alternat Med. 2021;2021:6634837. 10.1155/2021/6634837 PMC8081598 33995548

[80] Tarricone E, Mattiuzzo E, Belluzzi E, Elia R, Benetti A, Venerando R, et al. Anti-Inflammatory Performance of Lactose-Modified Chitosan and Hyaluronic Acid Mixtures in an In Vitro Macrophage-Mediated Inflammation Osteoarthritis Model. Cells. 2020;9(6):1328. 10.3390/cells9061328 PMC7349682 32466461

[81] Lee H, Kim H, Seo J, Choi K, Lee Y, Park K, et al. TissueGene-C promotes an anti-inflammatory micro-environment in a rat monoiodoacetate model of osteoarthritis via polarization of M2 macrophages leading to pain relief and structural improvement. Inflammopharmacology. 2020;28(5):1237–52. 10.1007/s10787-020-00738-y PMC7524813 32696209

[82] Xu Y, Wang JY, Zou Y, Ma XW, Meng T. Role of IL-1 Family Cytokines IL-36, IL-37, IL-38 in Osteoarthritis and Rheumatoid Arthritis: A Comprehensive Review. J Inflamm Res. 2024;17:4001–16. 10.2147/JIR.S474879 PMC11195677 38915806

[83] Hu H, Yang B, Li Y, Zhang S, Li Z. Blocking of the P2X7 receptor inhibits the activation of the MMP-13 and NF-kappaB pathways in the cartilage tissue of rats with osteoarthritis. Int J Mol Med. 2016;38(6):1922–32. 10.3892/ijmm.2016.2770 27748894

[84] Zheng S, Ren J, Gong S, Qiao F, He J. CTRP9 protects against MIA-induced inflammation and knee cartilage damage by deactivating the MAPK/NF-kappaB pathway in rats with osteoarthritis. Open Life Sci. 2020;15(1):971–80. 10.1515/biol-2020-0105 PMC7874593 33817283

[85] Charlier E, Relic B, Deroyer C, Malaise O, Neuville S, Collee J, et al. Insights on Molecular Mechanisms of Chondrocytes Death in Osteoarthritis. Int J Mol Sci. 2016;17(12):2146. 10.3390/ijms17122146 PMC5187946 27999417

[86] Riegger J, Schoppa A, Ruths L, Haffner-Luntzer M, Ignatius A. Oxidative stress as a key modulator of cell fate decision in osteoarthritis and osteoporosis: a narrative review. Cell Mol Biol Lett. 2023;28(1):76. 10.1186/s11658-023-00489-y PMC10541721 37777764

[87] Yang J, Hu S, Bian Y, Yao J, Wang D, Liu X, et al. Targeting Cell Death: Pyroptosis, Ferroptosis, Apoptosis and Necroptosis in Osteoarthritis. Front Cell Dev Biol. 2021;9:789948. 10.3389/fcell.2021.789948 PMC8804296 35118075

[88] Zhang J, Qi B, Liu M, Zhao T, Tan L. Influence of swimming exercise on the expression of apoptotic gene caspase-3 in chondrocytes in osteoarthritis. Am J Transl Res. 2021;13(4):2511–7. PMC8129308 34017409

[89] Zhu P, Wang Z, Sun Z, Liao B, Cai Y. Recombinant platelet-derived growth factor-BB alleviates osteoarthritis in a rat model by decreasing chondrocyte apoptosis in vitro and in vivo. J Cell Mol Med. 2021;25(15):7472–84. 10.1111/jcmm.16779 PMC8335691 34250725

[90] Mohetaer D, Cao L, Wang Y. Oxymatrine Protects Chondrocytes against IL-1beta-triggered Apoptosis in Vitro and Inhibits Osteoarthritis in Mice Model. Evid Based Complement Alternat Med. 2022;2022:2745946. 10.1155/2022/2745946 PMC9532098 36204118

[91] Zhou Z, Zhang L, Liu Y, Huang C, Xia W, Zhou H, et al. Luteolin protects chondrocytes from H2O2-induced oxidative injury and attenuates osteoarthritis progression by activating AMPK‐Nrf2 signaling. Oxidative Med Cell Longev. 2022;2022(1):5635797. 10.1155/2022/5635797 PMC8825676 35154568

[92] Jin H, Yang Y, Lei G, Zeng C, He K, Wang Y, et al. Pain intensity and trajectory following intra-articular injection of mono-iodoacetate in experimental osteoarthritis: a meta-analysis of in vivo studies. Cartilage. 2023;14(1):86–93. 10.1177/19476035221144748 PMC10076896 36628407

[93] Sasaki Y, Kijima K, Yoshioka K. Validity evaluation of a rat model of monoiodoacetate-induced osteoarthritis with clinically effective drugs. BMC Musculoskelet Disord. 2024;25(1):975. 10.1186/s12891-024-08083-9 PMC11605887 39609755

[94] Maleitzke T, Weber J, Hildebrandt A, Dietrich T, Zhou S, Tsitsilonis S, et al. Standardized protocol and outcome measurements for the collagen antibody-induced arthritis mouse model. STAR protocols. 2022;3(4):101718. 10.1016/j.xpro.2022.101718 PMC9519592 36152302

[95] Kong J-S, Jeong GH, Yoo S-A. The use of animal models in rheumatoid arthritis research. J Yeungnam Med Sci. 2023;40(1):23–9. 10.12701/jyms.2022.00773 PMC9946911 36411592

